# The BACHD Rat Model of Huntington Disease Shows Specific Deficits in a Test Battery of Motor Function

**DOI:** 10.3389/fnbeh.2017.00218

**Published:** 2017-11-03

**Authors:** Giuseppe Manfré, Erik K. H. Clemensson, Elisavet I. Kyriakou, Laura E. Clemensson, Johanneke E. van der Harst, Judith R. Homberg, Huu Phuc Nguyen

**Affiliations:** ^1^Donders Institute for Brain, Cognition and Behaviour, Department of Cognitive Neuroscience, Radboud University Medical Center, Nijmegen, Netherlands; ^2^Noldus Information Technology BV, Wageningen, Netherlands; ^3^Institute of Medical Genetics and Applied Genomics, University of Tübingen, Tübingen, Germany; ^4^Centre of Rare Diseases, University of Tübingen, Tübingen, Germany

**Keywords:** Huntington disease, polyglutamine disease, model characterization, motor function, transgenic rat, neurodegenerative disorders, fine motor control, automated home-cage monitoring

## Abstract

**Rationale**: Huntington disease (HD) is a progressive neurodegenerative disorder characterized by motor, cognitive and neuropsychiatric symptoms. HD is usually diagnosed by the appearance of motor deficits, resulting in skilled hand use disruption, gait abnormality, muscle wasting and choreatic movements. The BACHD transgenic rat model for HD represents a well-established transgenic rodent model of HD, offering the prospect of an in-depth characterization of the motor phenotype.

**Objective**: The present study aims to characterize different aspects of motor function in BACHD rats, combining classical paradigms with novel high-throughput behavioral phenotyping.

**Methods**: Wild-type (WT) and transgenic animals were tested longitudinally from 2 to 12 months of age. To measure fine motor control, rats were challenged with the pasta handling test and the pellet reaching test. To evaluate gross motor function, animals were assessed by using the holding bar and the grip strength tests. Spontaneous locomotor activity and circadian rhythmicity were assessed in an automated home-cage environment, namely the PhenoTyper. We then integrated existing classical methodologies to test motor function with automated home-cage assessment of motor performance.

**Results**: BACHD rats showed strong impairment in muscle endurance at 2 months of age. Altered circadian rhythmicity and locomotor activity were observed in transgenic animals. On the other hand, reaching behavior, forepaw dexterity and muscle strength were unaffected.

**Conclusions**: The BACHD rat model exhibits certain features of HD patients, like muscle weakness and changes in circadian behavior. We have observed modest but clear-cut deficits in distinct motor phenotypes, thus confirming the validity of this transgenic rat model for treatment and drug discovery purposes.

## Introduction

Huntington disease (HD) is a severe autosomal dominant neurological disorder. Typically, onset of symptoms is during middle age and it consists of a triad of motor, cognitive and psychiatric symptoms (Walker, 2007). The motor symptoms of HD are varied and encompass involuntary movements such as chorea as well as impaired voluntary movements, which cause limb incoordination and impaired hand function (Novak and Tabrizi, 2010). These symptoms are worsened by loss of postural reflexes and their pattern tends to change over time, with chorea declining and dystonia, rigidity and bradykinesia becoming more marked (Novak and Tabrizi, 2010; Nguyen and Cenci, 2015). Motoric symptoms are frequently involved in the cause of death, along with complications such as dysphagia and aspiration (Walker, 2007; Nguyen and Cenci, 2015). Typical latency from diagnosis to death is 20 years (Watt, 1990; Walker, 2007). The disease is caused by an expanded CAG mutation in the huntingtin (*HTT*) gene (Huntington’s Disease Collaborative Research Group, 1993), leading to initial atrophy and cell loss in the neostriatum (caudate nucleus and putamen in humans), then spreading to cortical areas and eventually affecting the whole brain (Vonsattel et al., 1985). Although disease progression is currently untreatable, efforts are aimed at identifying novel symptomatic, neuroprotective and reparative treatments (Frank, 2014). The latest drug approved for use in the United States, deutetrabenazine, adds to the other drugs that can alleviate some of the choreic and psychiatric symptoms of HD (Frank, 2014; Huntington Study Group et al., 2016). Part of that research effort involves identifying suitable animal models that provide a valid representation of the pathological and behavioral profile of the human disease and that can serve for the identification of therapeutic candidates and novel approaches to therapy (Kumar et al., 2010; Ross and Tabrizi, 2011; Pouladi et al., 2013). Between mammalian species, rats were the first animals used for scientific purposes and have been considered for decades as a key model organism in biomedical science, including neurological disorders and behavioral neuroscience (Cenci et al., 2002; Homberg et al., 2017). Accordingly, some complex behaviors and physiological processes that can be readily studied in rats are difficult or impossible to investigate in mice (Ellenbroek and Youn, 2016; Homberg et al., 2017). Comparative analyses of movements in rats and primates show homology of many motor patterns across species. Advances have been made in identifying rat equivalents of akinesia, tremor, postural deficits and dyskinesia, which are relevant to neurological disorders (Cenci et al., 2002).

The BACHD rat is a recently generated transgenic rat model expressing full-length human mutant huntingtin (mHTT) and is currently being characterized in order to understand its advantages and limitations concerning modeling of HD. BACHD rats present a wide range of behavioral abnormalities reminiscent of the cognitive, emotional and motor alterations observed in HD patients (Yu-Taeger et al., 2012; Abada et al., 2013a,b; Jansson et al., 2014; Clemens et al., 2015; Manfré et al., 2016; Clemensson et al., 2017a,b). Previous characterization studies in these rats showed the presence of emotional alterations, as suggested by a decreased anxious-like behavior in the elevated plus maze (Yu-Taeger et al., 2012). BACHD rats also exhibited associative memory deficits in a fear conditioning setup, impairments of their reversal learning performance in a cross maze task (Abada et al., 2013a) as well as deficits in prepulse inhibition (Abada et al., 2013b). Furthermore, BACHD animals exhibited signs of fronto-striatal impairment in different Skinner box tasks for short term memory (Clemensson et al., 2017b) and an impulsive-like phenotype was shown in a delayed discounting paradigm and in the Differential Reinforcement of Low Rate of Responding task (Manfré et al., 2016).

Although patients and several HD mouse models have been found to present cognitive symptoms already before the start of any motor symptoms (Carter et al., 1999; Paulsen et al., 2001; Van Raamsdonk et al., 2005; Lichter and Hershey, 2010), this could not be demonstrated for the BACHD rat. Instead, BACHD rats have repeatedly been found to show early impairments in the Rotarod test (Yu-Taeger et al., 2012; Abada et al., 2013b; Clemens et al., 2015) which progressively worsens over time (Yu-Taeger et al., 2012; Abada et al., 2013b; Clemens et al., 2015). Altered activity in the PhenoMaster (TSE Systems, Germany) and in an open field test-like setup as well as abnormalities in unhindered walking gait have also been reported (Yu-Taeger et al., 2012; Abada et al., 2013b). These findings suggest that a more in-depth investigation of various aspects of motor function in BACHD rats is needed. Therefore, the aim of this study was to further dissect the motor phenotype of this transgenic rat model of HD by combining traditional and modern paradigms. We hypothesize that this impairment might be due to deficits in: (1) fine motor control; (2) muscle strength and/or endurance; and (3) locomotor activity.

One of the fine motor functions that is possible to investigate is skilled reaching (the conventional term for the reach-to-eat act), which is a form of prehension in which a hand is used to grasp a food item and place it into the mouth for eating (Alaverdashvili and Whishaw, 2013). Skilled reaching is an everyday activity for humans (Sacrey and Whishaw, 2010). As mentioned earlier, rats serve as excellent models to reproduce deficits in motor ability, such as the ability to manipulate or reach various objects (Klein and Dunnett, 2012). Furthermore, manual dexterity is also a central daily activity and it is commonly disrupted by nervous system damage, often with permanent effects (Iwaniuk and Whishaw, 2000). Rodents use their forepaws in dexterous ways that are in some capacities homologous to humans (Iwaniuk and Whishaw, 2000; Cenci et al., 2002). Since HD patients have difficulties in manipulating and reaching objects, translating such behaviors to an animal model is of great interest, providing more information on a potential read-out for future treatments (Klein et al., 2012).

Another cause that might underlie the motor deficits of BACHD animals is an impairment in gross motor function. HD patients and animal models of HD present signs of peripheral motor pathology, including gait abnormality and muscle wasting (de Aragão et al., 2016). Hence, characterizing this aspect in rodent models of progressive neurodegenerative and muscle wasting diseases requires a battery of tests. Classical behavioral assays such as the holding bar and the grip strength test are still of high value to assess muscle function and coordination (Brooks and Dunnett, 2009; Klein et al., 2012; Nguyen and Cenci, 2015).

A further aspect that can be evaluated concerns altered locomotor activity, since HD patients exhibit imbalance, trouble in walking, clumsiness and unsteadiness (Di Maio et al., 1993). In previous studies, motor phenotypes in rodent models of HD and Duchenne muscular dystrophy have been assessed using different setups, showing consistent results (Hara et al., 2002; Hickey et al., 2008). Accordingly, in the last decade there has been a concerted effort towards automating methods for continuous automated home-cage assessment and for measuring motor function (Vandeputte et al., 2010; Chort et al., 2013; Bains et al., 2017). Such technologies are aimed at capturing a wider range of behaviors and are free from experimenter bias through the possibility to house rodents in automated home-cage environments for extended periods of time and to measure voluntary activity without interference from the investigator (Schaefer and Claridge-Chang, 2012). Here, BACHD and control rats were tested in an instrumented home-cage (PhenoTyper^®^, Noldus Information Technology) to monitor locomotor and circadian activity over time.

In the present study, we focused on tasks that may reveal subtle motor disturbances characteristic of human HD. To expand the repertoire of meaningful motor function tests, we combined classical behavioral paradigms with automated home-cage observations which allow high-throughput testing. The tests were performed at three different ages (2,7 and 12 months) to assess the onset and progression of specific motor symptoms and provide read-out parameters for future pre-clinical studies applying novel drugs for the treatment of HD.

## Materials and Methods

### Animals

Wild-type (WT) and transgenic (hemizygous BACHD; TG5 line) male rats carrying the mutant human HTT gene, under the control of the human HTT promoter and its regulatory elements were used. The transgene contained 97 CAG-CAA mix repeats, and additional 20 kb upstream and 50 kb downstream sequences ensured stability of the repeat length (Yu-Taeger et al., 2012). The construct has previously been used to generate the BACHD mouse (Gray et al., 2008). All animals were maintained on Sprague-Dawley background and genotyped according to previously published protocols (Yu-Taeger et al., 2012). As the study was a collaborative effort, two different cohorts have been used at University of Tuebingen (Tuebingen, Germany) for *Experiment 1*, and at Radboudumc (Nijmegen, Netherlands) for *Experiment 2*. Each group of animals was subjected to a different battery of tests, as described in the study design section.

For *Experiment 1*, 12 BACHD rats and 12 WT rats were obtained from in-house breeding with hemizygous BACHD males from the TG5 line (Yu-Taeger et al., 2012) paired with WT females (Charles River, Germany). Rats were weaned at 21 days of age and housed in genotype-matched groups of three rats per cage in type IV cages (38 × 55 cm) with high lids (24.5 cm from cage floor), containing wooden houses, nesting paper and wooden bedding material. All rats used for testing were handled on a daily basis. The animal facility kept 21–23°C, 55%–10% humidity, and was set to a partially reversed light/dark cycle with lights on/off at 02:00/14:00 during summer, and 01:00/13:00 during winter. Food and water regimen is described in the study design section. All experiments were approved by the local ethics committee (Regierungspraesidium Tuebingen) and carried out in accordance with the German Animal Welfare Act and the guidelines of the Federation of European Laboratory Animal Science Associations, based on European Union legislation (Directive 2010/63/EU).

For *Experiment 2*, 15 transgenic males were supplied from the original BACHD colony of Charles River (Wilmington, MA, USA) and an in-house breeding colony was preserved and maintained at Radboudumc (Nijmegen, Netherlands) by cross-breeding these males with WT female rats (Charles River, Germany). WT and BACHD animals (*n* = 12/group) were weaned at 21 days of age and group-housed two per cage with littermates of the same genotype in type IV cages (38 × 55 cm) containing plastic houses, nesting paper and wooden bedding material. Cages were in a constant temperature-humidity room (19.5°C—54% humidity) with a regular 12 h light/dark cycle with lights on/off at 8:00/20:00). Food and water were provided *ad libitum*.

Body weights were measured regularly throughout the study in both groups, and as seen with other cohorts of male BACHD rats, there was no difference in body weight between the genotypes (data not shown). It should be noted though that despite the unchanged body weight, BACHD rats have been reported to have a reduced bone and muscle mass and an increased fat mass from 3 months of age onwards (Jansson et al., 2014) but these parameters could not be assessed in the present study. All experiments were positively evaluated by the Animal Ethics Committee (“RU-DEC”, Nijmegen, Netherlands) and performed under a project license from the Central Committee on Animal Experiments (CCD, The Hague), in full compliance with the legal requirements of Dutch legislation on the use and protection of laboratory animals.

### Study Design

There were two experiments conducted for this study.

#### Experiment 1: Fine Motor Control

Prior to behavioral testing described below, all animals (2-month aged) were subjected to the Rotarod test (for detailed protocol see Clemens et al., 2015). This was done to ensure that BACHD rats exhibited the strong phenotype observed in previous studies (Yu-Taeger et al., 2012; Abada et al., 2013b; Clemens et al., 2015) and to avoid having a group of rats not being representative for the model. After confirming the presence of the Rotarod impairment (data not shown), animals were divided in two groups (*n* = 6/group) and longitudinally tested at 2, 7 and 12 months of age according to the following scheme. On any given day, one group was challenged with the pasta handling and the pellet reaching test, while the rats of the other group rested. Therefore, groups were assessed on alternating days until stable performance (10 sessions/age point for the pasta handling and eight sessions/age point for the pellet reaching test). Unless otherwise noted, behavioral testing took place during the light phase to allow optimal visualization of fine movements.

During non-testing periods, all animals were given access to food and water *ad libitum*. Two weeks before each testing phase, daily food amount was progressively reduced until rats reached 85% of their respective free-feeding body weights. Afterwards, they were fed a daily ration in order to maintain this restriction level, taking normal growth into consideration. The animals were weighed every morning to assess food restriction levels and fed in their home-cages at 17:00 h. Following the testing periods, animals were again fed *ad libitum*.

During scoring of both behavioral tests, the experimenters were blind to the rats’ genotypes, while this was not the case when the videos were gathered. After being tested at 12 months of age, all rats were used for additional food consumption tests published elsewhere (Clemensson et al., 2017a).

#### Experiment 2: Gross Motor Function and Locomotor Activity

Animals were divided in two groups of WT and BACHD (*n* = 6/group) and longitudinally tested at 2, 7 and 12 months of age according to the following scheme. On day 1, rats were challenged with the holding bar test and subsequently housed in the PhenoTyper^®^ cages for six consecutive days (day 1–6) to assess locomotor activity (de Visser et al., 2006). Animals were then taken out of the PhenoTyper^®^ and socially housed for 48 h (day 7–8) as resting period. On days 9–10, animals were, respectively, trained and tested for grip strength. All behavioral tests were performed during the light phase and carried out by a single experimenter, while another experimenter (blind to the rats’ genotype) was videotaping or scoring the behavior.

### Behavioral Procedures

#### Experiment 1: Tests for Fine Motor Control

##### Pasta handling test

The pasta-handling test was used to evaluate forepaw dexterity in the BACHD rats. This test has been found to be sensitive to a wide range of injuries and impairments (Allred et al., 2008; Tennant et al., 2010). Although the original protocols suggest using 7 cm-long spaghetti pieces, we have found that our rats eat in a hunched-over position, causing them to frequently break pasta pieces of such lengths. For the current study, the rats were therefore given strands of uncooked spaghetti (1.5 mm in width and 0.15 g/piece, Barilla, Italy) that were cut to lengths of 5 cm. Prior to the first test occasion, rats were habituated to the pasta by placing eight pieces into their social home-cages during five consecutive days. Afterwards, rats were given three habituation sessions in the test setup that was used. The test used a glass cage (28.5 × 29 × 29.5 cm) with mirrors on the floor and along two walls, which ensured a good view of the test animals. The sessions followed a similar structure during both, habituation and testing. At the start of each session, a rat was placed inside the setup. Afterwards, a single piece of spaghetti was dropped into the cage, and the rat was allowed to consume it. When the rat consumed the first piece, a new spaghetti piece was dropped into the cage. During habituation sessions, no video recordings were made, and the sessions ended either after consumption of two spaghetti pieces, or when 5 min had elapsed. Rats that did not consume the first pasta piece within the set time limit were given an additional session at the end of the day, during dark phase, to promote habituation. After the habituation sessions, all rats were given ten test sessions, organized as described in the study design section. For these sessions, pasta pieces were filed down to achieve blunt edges and marked with an ultrafine tip marker at specific intervals (1 cm increments) in order to facilitate visualization of the movement of the pasta strand during eating. During each session, rats were given between two and five pasta pieces, depending on their behavior, with the aim to obtain two consumption videos of good quality from each rat. Sessions during which a rat broke the pasta piece were excluded and not counted as consumption video. A minimum of 20 consumption videos was gathered for each rat and test age. For occasions where the initial ten testing sessions were insufficient to achieve this, additional test sessions were given. Rats were videotaped with a handheld camera (Sony HD Handycam, Japan) positioned to optimize the view of paw movements. Several behavioral parameters were scored from the videos. Some of these concerned detailed scoring of biting and chewing behavior, and are further described in the Supplementary Material (Figures S1 and S2). The primary readouts, however, concerned the rats’ forepaw use. For this, the total time spent actively handling the pasta piece was measured using a stopwatch during normal speed playback of the videos (MacLaren et al., 2014). This parameter specifically excluded occasions where the rats chewed, flipped over or dropped the pasta piece to obtain the time they actively manipulated it with their forepaws. Slow motion video playback (~50% of real-time) was then used to quantify the number of forepaw adjustments. The total number of adjustments of each paw was counted per trial. A normal adjustment was defined as any distinct removal and replacement of the paw, or of any number of digits, on the pasta piece after eating commences.

##### Pellet reaching test

To assess skilled reaching, the rats were assessed in a pellet reaching test. Other protocols of such behaviors are sensitive to discreet neuropathologies (Farr and Whishaw, 2002; Klein and Dunnett, 2012). All sessions were conducted in a transparent Plexiglas box (35 cm long × 35 cm wide × 35 cm tall) placed on a table surface. Each side presented two 1 cm wide, slit vertical openings that allowed the animals to reach for 45 mg grain-based precision pellets (Bio-Serv, Dustless Precision Pellets F0021, purchased through Bilaney Consultants, Duesseldorf, Germany) placed on a wooden frame attached to each side of the box, and allocated 3 cm above the floor. Before the first test occasion, the rats were habituated to the pellets by placing a spoonful into their home-cages for five consecutive days. Animals were then positioned in the apparatus individually and first acclimatized to the chamber with two shaping sessions in the dark phase, during which 20 pellets were placed, one at a time, on the wooden frame, at a distance of 1.5 cm from the openings. After shaping, limb preference of individual animals was determined by challenging each rat with a single session during which 100 reaching attempts were scored. During this, the pellets were placed centrally in front of one opening to allow the rat to use either paw. When 80% out of 100 reach attempts were made with one limb during this single session, that limb was identified as the preferred one. In the subsequent training sessions, food pellets were located contralateral to this limb at a distance of 1.5 cm from the slit openings. This enabled the animals to reach for pellets with their preferred limb. Animals that were not attempting to reach the pellets were further trained during the dark phase, placing the pellets at 1 cm distance from the slit openings to encourage reaching attempts. Some animals used their tongue instead of their paws and were further trained, during the dark phase, to reach the pellets at a distance of 2 cm from the openings. When all animals had made 20 reaching attempts within a single session, with the pellet placed 1.5 cm away from the slit, they progressed to the testing sessions. It took a total of 5 days for all rats to reach this criterion. During test sessions, rats were allowed to reach for a total of 30 pellets (considered as 30 separate trials). The first ten pellets were considered as warm up trials, and the following 20 were considered testing trials. The pellets given on subsequent trials were placed so that the pellet position alternated between two sides of the box, but randomized between the two available slits on each wall. Through this, a predictable pattern of side alternation, but unpredictable pattern of specific pellet position was obtained. This was done to induce a partial searching behavior among the rats, which ensured goal-directed reaches. It should be noted that the exact use of the different slits was still balanced so that no opening was overrepresented during testing. Rats were given one test session per day until they reached a stable performance. This resulted in a total of eight test sessions per age point. The success rate of the rats’ reaching attempts was used as outcome measure. Reaches were considered successful if the rat had grabbed the pellet on the first reach attempt and managed to retrieve and eat it without dropping it into the cage bedding. All other behaviors were considered failure. Success rates were calculated as the number of successful reaches out of the final 20 pellets offered at each session. Recordings were made with a small action camera (Mini WiFi Camcorder 1, Rollei, Germany) connected to a computer using AVS videorecorder^1^. Slow motion video playback (~50% of real-time) was used to calculate the number of successful reaches.

#### Experiment 2: Tests for Gross Motor Function and Locomotor Activity

##### Holding bar test

The holding bar test was used to assess forelimb hanging strength and balance over time (Li et al., 2004; van Putten et al., 2012). The test apparatus consisted of a 37 cm-wide and 3 mm-thick wooden bar tightly secured between two vertical stands placed around 75 cm above a pillow. The height was sufficient to encourage the animals to hold the bar, but also low enough to prevent them from injuries when falling down. Each animal was handled via the body and brought near the bar, allowing the grasping of the bar with the forepaws only. Each rat was videotaped with an iPhone^®^ 6S (Apple, Cupertino, CA, USA) and given three consecutive trials (ITI = 1 min). The average of the three trials was used as main outcome measure. Moreover, the Holding Impulse (s*g) = hang time (s) × body mass (g) was used to correct for the negative effects of body mass on the hanging time, and it reflected the minimal amount of sustained tension (impulse) that the animal developed for supporting itself on the bar against gravity for the longest period of time (van Putten et al., 2012). When improper behavior occurred (e.g., balancing on top of the bar or deliberately jumping off the bar) the trial was omitted and repeated. Suspension latencies were scored with slow motion video playback (~50% of real-time) using The Observer XT 12 (Noldus Information Technology, Wageningen, Netherlands).

##### Grip strength

This test is based on the tendency of a rat to instinctively grasp a bar or a grid when suspended by the body, and permits assessment of the strength of the forelimbs. The test apparatus (Grip Strength Meter, Ugo Basile, Italy) consisted of a grasping bar attached to a force transducer in order to measure the maximum force applied by the rat during the pull. The unit of force used was grams-of-force. Each animal was handled via the body and brought near the bar, allowing the grasping of the grid with both forepaws and then gently pulled back until they released it. Animals were trained and tested on two consecutive days, using the same protocol. Five such measurements were obtained for each animal, and the resting period between each pull was 1 min (Jeyasingham et al., 2001; Aartsma-Rus and van Putten, 2014).

##### Locomotor activity in the PhenoTyper

Locomotor activity was recorded by videotracking in the PhenoTyper (Noldus Information Technology, Wageningen, Netherlands), an instrumented home-cage in which rodent behavior was automatically monitored through a video-based observation system, as described in detail by de Visser et al. (2006). The cages (45 cm × 45 cm × 45 cm) were made of transparent Perspex walls with an opaque Perspex floor covered with cellulose-based bedding (Cellu-Dri, LBS Biotechnology, United Kingdom), and equipped with a water bottle, a feeding station and a shelter in one corner (14.3 cm × 14.3 cm × 11.5 cm). Food and water were provided *ad libitum*. Video tracking was performed by an infrared-sensitive video camera installed in the top unit of each cage, infrared lighting sources and hardware and software needed for videotracking. EthoVision 9 was used for data acquisition and Ethovision XT 11.5 for analysis (Noldus Information Technology, Wageningen, Netherlands). Rats were introduced into the PhenoTyper during the light phase (between 12:00 h and 16:00 h) and monitored for six consecutive days. Spontaneous locomotor activity was assessed, scoring the following parameters: distance moved, velocity, number of jumps and time spent on top of the shelter. We additionally investigated potential sleep disruption and/or altered circadian rhythmicity, taking into account the time spent inside the shelter. All parameters were calculated using the means of day 4–6 (de Visser et al., 2006) and results were then split into dark and light phases, according to the day-night cycle of the animals. No human interference took place between the start and the end of the observations (hence, no intermediate cleaning of the cages).

### Statistical Analyses

All statistical analyses were conducted using GraphPad Prism v.6.0 (GraphPad Software, San Diego, CA, USA). Two-way repeated measures ANOVAs were used to analyze all parameters. Age was used as within-subject factor, and genotype as between-subject factor. Bonferroni *post hoc* test was used to follow up any significant effect of genotype found in the two-way ANOVAs. A *p*-value < 0.05 was considered statistically significant.

During *Experiment 1*, two BACHD rats did not consume the pasta strands during every testing day, and were therefore excluded from the analysis. Between two testing periods of *Experiment 2*, two BACHD rats fell ill and had to be sacrificed and removed from the experiment at 9 and 11 months of age, respectively. In both cases, the illnesses concerned tumors. Due to technical problems, the locomotor activity/circadian rhythmicity data from two WT and four BACHD rats could not be analyzed.

Thus, the n of the analyses changed as follows: for the pasta handling test (WT: 12, BACHD: 10), for the pellet reaching task (WT: 12, BACHD: 12), for the holding bar test (WT:12, BACHD: 10), for the locomotor activity/circadian rhythmicity (WT: 8, BACHD: 8), for the grip strength (WT:12, BACHD: 10). Age development analyses excluded data from animals that were not assessed at all ages. No other exclusion criteria were used.

## Results

### Experiment 1: Fine Motor Control

#### Pasta Handling Test

**Figure 1:**
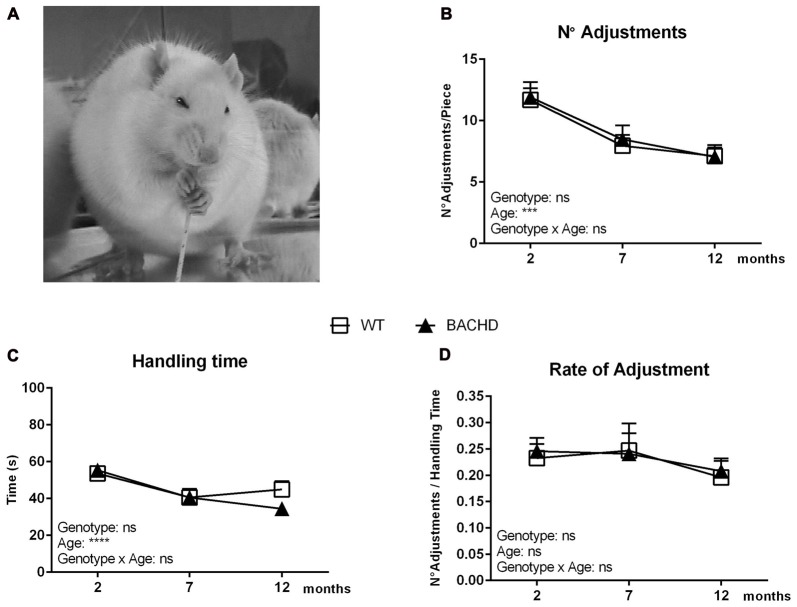
Basic parameters of the pasta handling test. **(A)** A photo displaying a rat during consumption of a spaghetti piece. Rats grasp the pasta pieces with both front paws and guide them using a coordinated asymmetrical pattern. The “grasp” paw holds the pasta in a whole-paw grasp, typically positioned lower (away from the mouth) on the piece at the start of eating. The other paw is named the “guiding” paw due to the fact that it is closer to the mouth than the grasping paw and is used to guide the pasta accurately between the teeth (Allred et al., 2008). **(B)** Age development of the number of adjustments needed for consuming a single spaghetti piece. **(C)** Age development of the total time spent handling a single spaghetti piece. **(D)** Age development of the rate of adjustment (number of adjustments over the total handling time). Data are expressed as means ± SEM. Two-way ANOVA results are displayed within each graph. Results from *post hoc* analysis are indicated in case significant genotype differences were found. ***p < 0.001, ****p < 0.0001, ns (not significant).

Figure 1A shows a photo of a rat during the pasta-handling test, displaying its typical paw placement during consumption. The number of adjustments required to eat the pasta piece was calculated over different sessions at the three different test ages (Figure 1B). All groups were found to use less adjustments by the final age of testing (age effect: *F*_(2,40)_ = 17.51, *p* < 0.0001) without any significant genotype or interaction effects. The handling time also changed with age (*F*_(2,42)_ = 16.08, *p* < 0.0001), with both WT and BACHD rats showing a longer handling time during the first test age compared to the following two (Figure 1C). There was no difference between WT and BACHD rats’ handling time at any age (genotype effect: *F*_(1,21)_ = 0.3779, *p* = 0.5454). The rate of adjustment, shown in Figure 1D, served as an indication of the number of adjustments over the total time of handling, which did not unravel significant age, genotype or interaction effects (age effect: *F*_(2,40)_ = 2.195, *p* = 0.1246; genotype effect: (*F*_(1,20)_ = 0.02155, *p* = 0.8848; interaction effect: *F*_(2,40)_ = 0.1206, *p* = 0.8867).

We further performed a detailed analysis of parameters related to biting and chewing, which did not reveal any prominent phenotypes, and are described and discussed in the Supplementary Material (Figures S1 and S2).

#### Pellet Reaching Test

**Figure 2:**
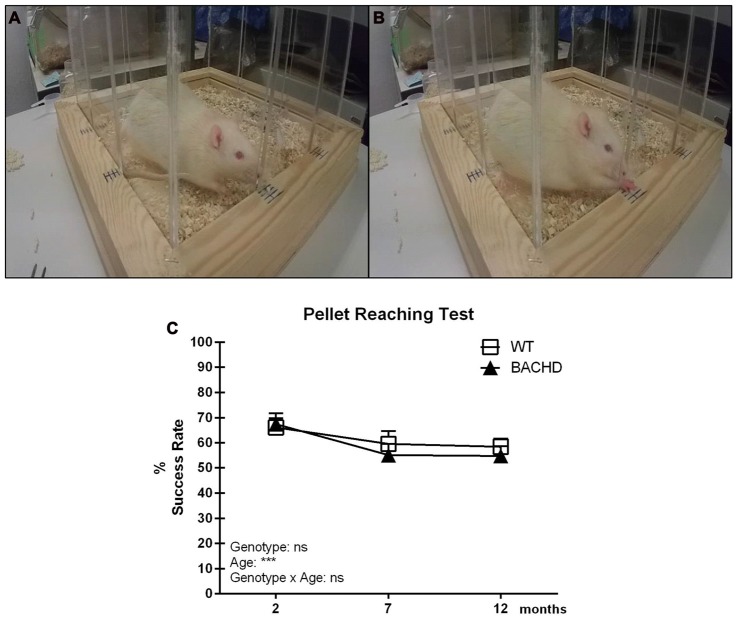
Pellet Reaching test. **(A)** Olfactory engagement with a food pellet and body posture of a rat before initiating a reach. **(B)** A rat reaching for a pellet through one of the slit openings. **(C)** Timeline of mean reaching success (per 20 reaches) in BACHD and wild-type (WT) control rats tested at 2, 7 and 12 months of age. Data are expressed as mean success rate over eight different sessions ± SEM. Two-way ANOVA results are displayed within each graph. Results from *post hoc* analysis are indicated in case significant genotype differences were found. ***p < 0.001, ns (not significant).

Figures 2A,B show the pellet reaching setup with an animal engaging in the test. Figure 2C illustrates the mean reaching scores in the pellet reaching test. Performance did not differ between the two groups at any age, although success rate of both genotypes dropped over time (age effect: *F*_(2,22)_ = 10.26, *p* = 0.0007; genotype effect: *F*_(1,11)_ = 0.2214, *p* = 0.6472). The major reason for failure to retrieve a pellet in either group was that pellets were either dropped during the reaching attempt(s) or displaced from the frame. It should be noted that the protocol and setup for the pellet reaching resulted in rats frequently standing at an angle to the openings rather than straight in front of them.

However, more specific scoring suggested that differences in body position did not affect the rats’ success rate, and did not differ between genotypes (data not shown).

### Experiment 2: Gross Motor Function and Locomotor Activity

#### Holding Bar

**Figure 3:**
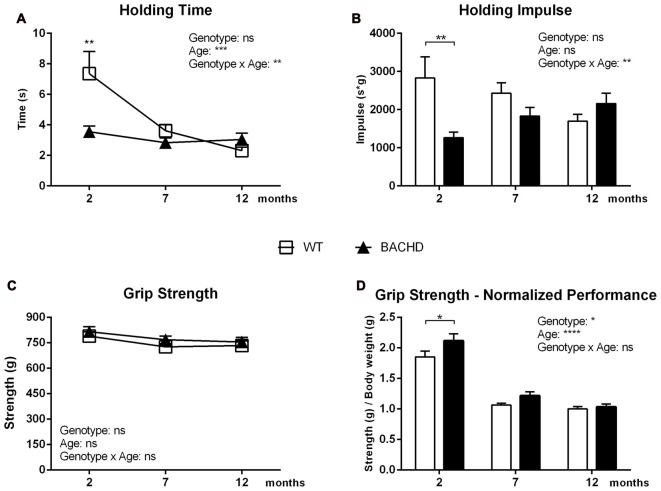
Holding Bar and Grip Strength tasks. **(A)** Mean latency in the Holding Bar maneuver. **(B)** The Holding Impulse, outcome measure (s*g) reflecting the minimal amount of sustained tension (impulse) that is needed to oppose gravity. **(C)** Performance in the Forelimb Grip Strength test.** (D)** Performance in the Forelimb Grip Strength test normalized for body weight. Data are expressed as means ± SEM. Two-way ANOVA results are displayed within each graph. Results from *post hoc* analysis are indicated in case significant genotype differences were found. *p < 0.05, **p < 0.01, ***p < 0.001, ****p < 0.0001, ns (not significant).

Figure 3A presents the average holding time of three trials, which reduced in both genotypes between 2 and 7 months of age, showing significant age (*F*_(2,40)_ = 10.06, *p* = 0.0003) and age × genotype effect (*F*_(2,40)_ = 6.226, *p* = 0.004). BACHD rats exhibited significant impairment compared to WT rats at 2 months of age only, as shown by the reduced average hanging time (genotype difference in *post hoc* analysis 2 months: *p* < 0.01). At 7 and 12 months of age, a genotype effect was not evident (*F*_(1,20)_ = 3.452, *p* = 0.078).

The holding impulse decreased in WT rats with increasing age, while it non-significantly increased in BACHD rats (age effect: *F*_(2,40)_ = 0.2972, *p* = 0.1106; genotype effect: *F*_(1,20)_ = 2.788, *p* = 0.1106; Figure 3B), resulting in a significant age × genotype effect (*F*_(2,40)_ = 7.318, *p* = 0.002). Although ANOVA revealed no overall genotype effect, *post hoc* analyses indicated the presence of deficits in the holding impulse in BACHD rats at 2 months of age (*p* < 0.01).

#### Grip Strength

Figure 3C represents the average of the three highest values of grip strength, which did not show any significant genotype (*F*_(1,20)_ = 3.861, *p* = 0.0635), age (*F*_(2,40)_ = 2.72, *p* = 0.0781) or interaction effects (*F*_(2,40)_ = 0.07663, *p* = 0.9264). Figure 3D shows the rats’ performance normalized to the animals’ individual body weight. The performance of BACHD and WT rats worsened between the age of 2 and 12 months, as reflected by a significant age effect (*F*_(2,40)_ = 123.1, *p* < 0.0001). The ANOVA revealed a general genotype effect (*F*_(1,20)_ = 6.186, *p* = 0.0218), but no significant genotype × age interaction. *Post hoc* analyses indicated that BACHD rats had significantly increased grip strength at 2 months of age (*p* < 0.05).

#### Locomotor Activity in the PhenoTyper

##### Dark phase

**Figure 4:**
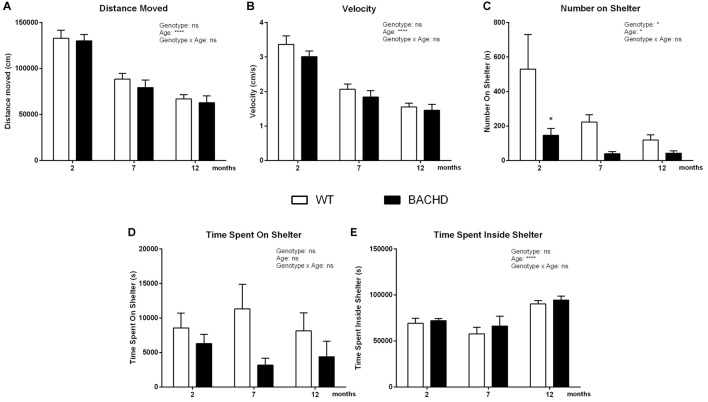
Home-cage assessment in the PhenoTyper—Active/dark phase activity. **(A)** Distance Moved. **(B)** Mean velocity. **(C)** Number of jumps onto the shelter. **(D)** Time spent on the shelter. **(E)** Time spent inside shelter. Data are expressed as means ± SEM. Two-way ANOVA results are displayed within each graph. Results from *post hoc* analysis are indicated in case significant genotype differences were found. *p < 0.05, ****p < 0.0001, ns (not significant).

Distance traveled (age effect: *F*_(2,32)_ = 134.5, *p* < 0.0001; Figure 4A) and velocity (age effect: *F*_(2,32)_ = 112.8, *p* < 0.0001; Figure 4B) during the dark phase was gradually reduced with age in animals of both genotypes. At each age, these parameters indicated similar behavior of WT and BACHD rats (distance traveled genotype effect: *F*_(1,16)_ = 0.35, *p* = 0.5624; velocity genotype effect: *F*_(1,16)_ = 1.101, *p* = 0.3095). However, BACHD rats showed a significant decrease of the number of jumps on top of the shelter compared to age-matched WT rats, exhibiting significant age (*F*_(2,32)_ = 3.886, *p* = 0.0308) and genotype differences (Figure 4C; *post hoc* analyses 2 months: *p* < 0.05). On the other hand, animals of different genotypes did not present any significant differences in the time spent on the shelter (genotype effect: *F*_(1,16)_ = 2.245, *p* = 0.1535; Figure 4D). Moreover, BACHD and WT rats did not spend different amounts of time inside the shelter (genotype effect: *F*_(1,16)_ = 0.7144, *p* = 0.4105); there was only a significant effect of age (*F*_(2,32)_ = 16, 77, *p* < 0.0001; Figure 4E).

##### Light phase

**Figure 5:**
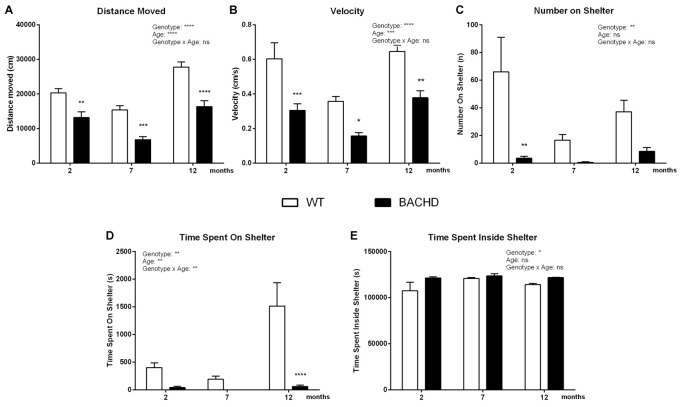
Home-cage assessment in the PhenoTyper—Inactive/light phase activity. **(A)** Distance Moved. **(B)** Mean velocity. **(C)** Number of jumps onto the shelter. **(D)** Time spent on shelter. **(E)** Time spent inside shelter. Data are expressed as means ± SEM. Two-way ANOVA results are displayed within each graph. Results from *post hoc* analysis are indicated in case significant genotype differences were found. *p < 0.05, **p < 0.01, ***p < 0.001, ****p < 0.0001, ns (not significant).

The overall locomotor activity changed with age in both WT and BACHD, showing a significant age effect in the distance moved (*F*_(2,32)_ = 45.45, *p* < 0.0001; Figure 5A), in the velocity (*F*_(2,32)_ = 11.47, *p* = 0.0002; Figure 5B) and in the time spent on the shelter (*F*_(2,32)_ = 7.809, *p* = 0.0017; Figure 5D). BACHD rats showed a significant reduction of the distance traveled and the velocity, exhibiting significant genotype differences in *post hoc* analyses for distance moved (2 months: *p* < 0.01, 7 months: *p* < 0.001, 12 months: *p* < 0.0001; Figure 5A), for velocity (2 months: *p* < 0.001, 7 months: *p* < 0.05, 12 months: *p* < 0.01; Figure 5B) and for number of jumps on top of the shelter (2 months: *p* < 0.01; Figure 5C). Moreover, BACHD rats showed decreased time spent on the shelter (Figure 5D), exhibiting a significant genotype effect in *post-hoc* analyses (12 months, *p* < 0.0001) and significant interaction between genotype and age (*F*_(2,32)_ = 6.750, *p* = 0.0036). On the contrary, BACHD rats spent significantly more time in the shelter, showing a genotype effect (*F*_(1,16)_ = 6.132, *p* = 0.0248) in the time spent inside the shelter (Figure 5E), without showing any age (*F*_(2,32)_ = 1.408, *p* = 0.2594) or interaction (*F*_(2,32)_ = 0.7329, *p* = 0.4884) effect.

## Discussion

In previous studies BACHD transgenic rats presented a strong motor coordination deficit in the Rotarod test (Yu-Taeger et al., 2012; Abada et al., 2013b; Clemens et al., 2015), mild gait impairments and altered open field activity (Abada et al., 2013b). The use of multiple motor paradigms in the present study allowed us to further investigate fine motor skills, muscle strength and endurance as well as several parameters of activity-related motor function in a home-cage-like environment. Taken together, these findings expand the existing knowledge of the behavioral phenotypes of the BACHD rat, showing the presence of selected behavioral deficits.

Regarding fine motor control, BACHD rats did not present any significant impairment. Although HD patients are significantly impaired in fine motor skills such as skilled reaching or manual dexterity (Klein et al., 2011), both parameters appeared to be intact in BACHD rats when challenged with the pasta handling and the pellet reaching tests. The results are, however, in line with previous observations in another rat model of HD (Fielding et al., 2012). Overall, these results suggest that fine motor aspects most likely did not contribute to the gross motor deficits seen in Rotarod performance or in the gait pattern.

Concerning muscle function, BACHD rats appeared significantly impaired in the holding bar task at 2 months of age, suggesting reduced muscle endurance. However, no differences were found at 7 and 12 months of age, as the WT rats’ performance dropped down to BACHD rat level at these ages. The difference at 2 months of age is possibly related to a reduced amount of muscle mass and increased amount of fat mass carried by BACHD rats, as found in a previous study (Jansson et al., 2014). A reduced performance in the holding bar test could therefore be expected, as the animals have to hold the same body weight with a significantly lower amount of muscle mass. On the other hand, the drop that WT animals exhibited might be either due to repeated testing or due to the large increase in body weight from 2 to 7 months of age, which might have made it impossible for the rats to hang longer than a certain minimum amount of time. Thus, the test might not allow us to draw final conclusions on muscle endurance in older rats.

Conversely, the grip strength test did not show an indication that BACHD rats’ “passive” forelimb grip strength was reduced, even at the youngest age. A possible explanation could be that in the holding bar test the rats have to work against gravity and their own weight, while this is not the case in the grip strength test. Due to the abnormal body composition of BACHD rats with reduced muscle and increased fat mass, workload during the holding bar test is therefore higher than that of WT rats. In contrast, WT and BACHD rats are likely exposed to a more comparable workload during the grip strength test.

Additionally, previous studies provided evidence of peripheral phenotypes such as weight loss and muscle wasting in HD patients (Cepeda et al., 2007; Imarisio et al., 2008; Miller and Bezprozvanny, 2010), progressive skeletal muscle atrophy in HD patients and in the R6/2 mouse model for HD (She et al., 2011; Zielonka et al., 2014). The impairment seen in the holding bar test might be also related to deficits in the joints, ligaments and tendons, all structures which are compromised in HD patients and in the BACHD mouse model (Nguyen and Cenci, 2015; de Aragão et al., 2016). Further studies would be required to investigate if the deficit we have reported is due to the reduced muscle mass or to a peripheral pathology or a functional impairment.

In addition to the assessment of motor phenotypes with classical tests, we implemented the observation in an automated home-cage system to investigate general aspects of motor function related to rats’ baseline behavior. In this setup, we found BACHD rats to show a generally reduced number of jumps on the shelter, while the time spent on the shelter was similar to WT rats. This suggests a specific motoric difficulty to reach the top of the shelter rather than a reduced motivation to use it. Interestingly, this phenotype was present at all investigated ages.

Furthermore, we found BACHD rats to have a generally reduced locomotor activity, specifically restricted to the light phase. These data suggest that, in our setting, the overall walking ability and activity of the rats was relatively preserved, as it was unaffected during the major activity phase. This is in line with previous observations in BACHD rats of the same age, which showed only mild gait abnormalities in both static and dynamic parameters during Catwalk testing (Abada et al., 2013b). Conversely, the results are in contrast with a lower rearing and ambulatory activity observed with the PhenoMaster system at 3 and 6 months of age (Yu-Taeger et al., 2012) and with an initial hyperactivity followed by hypoactivity starting at 4 months of age exhibited by BACHD rats in an open field test-like setup (Abada et al., 2013b). The different outcome could be due to the different test protocols and test setups used. Most prominently, we screened the animals for six consecutive days, while the other two studies screened for 22 h and 1 h, respectively. Thus, those results will probably rather reflect the rats’ response to novelty, while we have investigated their baseline behavior.

The reduction in activity during the light phase might suggest the existence of an altered circadian rhythm, which would be in line with previous studies in HD patients and in the BACHD mouse model (Morton et al., 2005; Kuljis et al., 2012). However, further and more detailed studies are necessary to investigate the mechanism underlying this behavioral alteration.

The robustness of our motor assessment study is that we performed a battery of behavior experiments, combining novel and classical test setups under well controlled environments (temperature, humidity, food restriction) making the monitoring of behavior across different ages possible. However, it has to be noted that we performed multiple behavioral tests with the same group of rats. Repeated testing might influence the outcome of the tests (carry-over effect), although such an approach offers a better possibility to investigate phenotype onset and development than in separate cohorts of rats. Another limitation is that we performed the classical tests during the light phase in order to enable better visualization of the rats’ behavior. However, it is known that behavioral readouts differ when assessed during the light or dark phase, and the dark phase still represents the natural activity phase of rats.

Taken together, our study revealed specific motoric impairments in the BACHD rats, which might be related to a reduced muscle mass and increased fat mass, as reported earlier (Jansson et al., 2014). We suspect that different factors might have influenced BACHD rats’ strong impairment in the Rotarod test reported in previous studies. Although there is definitely a motor component, the deficit may also be related to other, non-motor phenotypes such as motivation or anxiety, as such differences have been found early on Yu-Taeger et al. (2012), Jansson et al. (2014) and Clemensson et al. (2017b). Most importantly, the study revealed novel readouts that can be used for addressing the efficacy of novel therapies on different parameters of motor function in pre-clinical research.

## Author Contributions

GM, EKHC, EIK, LEC, JEH, JRH and HPN: conceived and designed the experiments. GM: performed the experiments. GM and EKHC: analyzed the data. GM, EKHC, EIK, LEC, JRH and HPN: wrote the manuscript. HPN: supply BACHD rat breeders and genotyping.

## Conflict of Interest Statement

At the time of the studies, the authors GM, EIK and JEH were working for the EU funded “PhenoRat” project of which Noldus Information Technology was an industrial partner. JEH was part-time scientific project advisor for “PhenoRat” employed by Noldus Information Technology. The other authors declare that the research was conducted in the absence of any commercial or financial relationships that could be construed as a potential conflict of interest.

## Footnotes

^1^http://www.avs4you.com/AVS-Video-Editor.aspx
