# Supplementary material for: The BACHD Rat Model of Huntington Disease Shows Specific Deficits in a Test Battery of Motor Function
Source: Front Behav Neurosci. 2017 Nov 3;11:218. doi: 10.3389/fnbeh.2017.00218 (PMC5675855; doi:10.3389/fnbeh.2017.00218)
Supplement: Supplementary file 1 [file Data_Sheet_1.docx]

Supplementary Material

The BACHD rat model of Huntington Disease shows specific deficits in a test battery of motor function

**Giuseppe Manfré^1,2,3^, Erik Karl Håkan Clemensson^3,4^, Elisavet I. Kyriakou ^1,2,3^, Laura Emily Clemensson^3,4^, Johanneke E. Van der Harst^1,2^, Judith R. Homberg^1^, Huu Phuc Nguyen^3,4*^**

^1^Donders Institute for Brain, Cognition and Behaviour, Department of Cognitive Neuroscience, Radboud University Medical Center, Nijmegen, The Netherlands

^2^Noldus Information Technology BV, Wageningen, The Netherlands

^3^Institute of Medical Genetics and Applied Genomics, University of Tübingen, Tübingen, Germany

^4^Centre of Rare Diseases, University of Tübingen, Tübingen, Germany

*** Correspondence:** Huu Phuc Nguyen

hoa.nguyen@med.uni-tuebingen.de

# Supplementary Data

## Material and methods

In addition to the parameters discussed in the main article, parameters relating to orofacial aspects of food consumption and consumption speed were scored from subsets of the videos obtained from the pasta handling test.

The full consumption time was measured from the point that a rat positioned the spaghetti piece into its mouths and started biting, to the point where the end of the pasta piece disappeared into its mouth. This parameter was scored at all ages. For videos gathered at two months of age, separate measurements of the time the rats spent actively biting on the pasta piece and the time they spent chewing and swallowing were also taken. The periods of active biting were clearly identifiable by the audible bite sounds that the rats produced. The periods of chewing were also clearly identifiable by the rat removing the spaghetti piece from its mouth and making characteristic chewing motions with their jaws. An additional and more discreet behavior was, however, included in these chewing periods. The rats would on occasion make breaks from biting on the spaghetti piece, and sit motionless with closed mouths, while still seemingly maintaining focus on the task at hand. The behavior was coupled with distinct sounds, and was deemed to be part of the feeding behavior, as we have not encountered it in other circumstances. Periods where the rats adjusted the position of the spaghetti piece, or stopped focusing on the spaghetti piece entirely, were not included in the measurement of consumption time, biting time or chewing time. Notably, these behaviors were all excluded despite the fact that rats would often couple them with some chewing motions. This was done to obtain a similar scoring protocol to what has been used in other studies of the BACHD rats’ food consumption behavior (Clemensson et al., 2017). The parameters above were scored using the open-source BORIS software for video annotation (Friard and Gamba, 2016). Videos gathered at 12 months of age were subjected to additional analyses to obtain the number of bites needed to consume a spaghetti piece and measure the temporal spacing of individual bites. This analysis was performed with The Observer XT 12.5 (Noldus Information Technology, Wageningen, The Netherlands), which allowed simultaneous viewing of a pasta handling video and its audio track. The timestamp of each audible bite sound was noted, and further processed to obtain the parameters of interest.

## Statistical analysis

Videos where the full feeding was not recorded, where the rats broke the pasta piece, or handled it with only one paw were excluded from the analysis of all parameters. For the analysis of consumption time, biting time and chewing time, average values were obtained for each test age and rat, during periods of stable performance. Ultimately, the data was for a given rat was based on an average of 15 videos (i.e. individual pasta piece consumptions) during the first test age, 11 videos during the second test age, and 5 videos during the final test age. Sound analysis was based on four trials per rat. The videos were selected to obtain a sample of videos with optimal audio quality, during a period of stable performance.

Age progression of consumption time was subsequently analyzed with a two-way ANOVA, using age as within-subject factor, genotype as between-subject factor and Sidak’s post-hoc test. One WT and three BACHD rats were excluded from the analysis, as data was not available for all ages. Thus the final n for this analysis was 11 WT rats and 9 BACHD rats. The analyses of consumption time, time spent biting and time spent chewing measured at the first test age used simple comparisons between WT and BACHD rats, and thus used individual t-tests. The n for these analyses were 12 for both genotypes. The total number of bites made during consumption of a spaghetti piece also used a simple comparison between WT and BACHD rats, but used a Mann-Whitney U test as the data from the BACHD rats did not appear to be normally distributed. Finally, data for frequency distribution of bite intervals were analyzed with two-way ANOVAs, using genotype as between-subject factor, bite interval as within-subject factor, and Sidak’s post-hoc test.

## Results

The time needed for consuming the spaghetti piece changed with age (Age effect: *p*<0.001), with both WT and BACHD rats showing a seemingly longer consumption time during the first test age compared to the following two (Supplementary Figure 1A). There was no difference between WT and BACHD rats’ consumption time at any age. Interestingly, most of the consumption time was made up of time spent actively biting on the spaghetti piece, while very little time was spent on chewing and swallowing behaviors (Supplementary Figure 1B, 1C, 1D).

Supplementary Figure 2A shows an example of the audio readout that was used to score individual bites. There was no significant difference between WT and BACHD rats in terms of the total number of bites that were needed in order to consume a full spaghetti piece (Supplementary Figure 2B) or in terms of the absolute or relative frequency distribution of bite intervals (Supplementary Figure 2C, 2D). Most bites were evenly spaced, with intervals around 0.11-0.20 seconds, although some bites appeared in much closer succession, showing intervals of 0.00-0.05 seconds. This is also evident from the audio printout shown in Supplementary Figure 2A. It should, however, be noted that this might not constitute individual biting motions, but rather a single bite causing the spaghetti piece to break in multiple places (e.g. inside the rats’ mouth). Although the analysis presented in Supplementary Figure 2 did not reveal any significant difference between WT and BACHD rats, it should be noted that one BACHD rat performed a remarkably high number of bites compared to the rest of the group. Excluding this rat from analysis did result in significant differences being detected between the genotypes (*p*<0.05 for Supplementary Figure 2B; Genotype effect: *p*<0.05, Interaction effect: *p*<0.01, post-hoc results for 0.00-0.05 bin: *p*<0.01, post-hoc results for 0.11-0.15 bin: *p*<0.05 for Supplementary Figure 2C; unchanged results for Supplementary Figure 2D). This analysis approach was, however, not considered to be appropriate, as there was no reason to assume that the outlier was caused by sub-optimal experimental conditions, but was likely just a representative of biologically based variation between rats.

## Discussion

There was no difference between WT and BACHD rats in terms of how much time they needed to consume the spaghetti pieces. This is in line with results from measuring consumption rate of small (45mg) reward pellets (Jansson et al., 2014), but in contrast with results from measuring consumption rate of single pellets of standard rodent chow (Clemensson et al., 2017). In the latter case, BACHD rats have been found to reliably require longer time than WT rats when consuming chow pieces of comparable size and shape. As discussed in (Clemensson et al., 2017), the reason for this discrepancy might be due to the different involvement of chewing behaviors in the three food consumption tests. Consuming the small reward pellets appears to involve very little chewing (Jansson et al., 2014), which is also true for the consumption of spaghetti pieces (Supplementary Figure 1D). In contrast, consumption of chow pieces, involves frequent, and extensive chewing (Supplementary Figure 8 in (Clemensson et al., 2017)). Thus, as a clear impairment in consumption rate has only been seen when assessing consumption of chow pieces, it is likely that BACHD rats show generally unimpaired biting and swallowing, while chewing motions are more strongly impaired. Still, discreet impairments have been noted also during biting behaviors (Clemensson et al., 2017). In line with this, the results from the current study show some indications that BACHD rats might suffer from discreet biting impairments, although the results are unclear (i.e. only significant if outliers are excluded). Essentially, although there does not appear to be any overt impairment present, BACHD rats might suffer from discreet deficits, resulting in them taking slightly fewer bites compared to WT rats. Unfortunately, the audio quality of the videos recorded in the current study was of generally poor quality, resulting in a quite limited data set (i.e. four spaghetti pieces per rat). A more extensive study is thus needed in order to determine if the slight trends noted in the current results are indicative of an actual phenotype, or due to chance.

# Supplementary Figures





**Supplementary Figure 1.** **Spaghetti consumption behaviors**

(A) Age development of the time needed to consume a spaghetti piece. Group mean plus standard error is indicated. Results from two-way ANOVA are displayed in bottom left corner of graph. Results from post-hoc test are displayed for data points where performance of WT and BACHD rats differed significantly. Detailed analysis of spaghetti consumption at two months of age is displayed in separate graphs for the full consumption time (B), the total time spent actively biting on the spaghetti piece (C), and the total time spent on chewing and swallowing (D). Graphs indicate performance of individual rats and group mean.

(***) *p* < 0.001, ns (not significant)





**Supplementary Figure 2. Bite sound analysis of spaghetti consumption**

(A) An example of the audio printout that was analyzed to obtain the data of interest. Note that the second and seventh bite sounds appear in very close succession of the previous bite sounds. (B) Basic comparison of the total number of bites needed to consume a single spaghetti piece. Performance of individual rats and group mean is indicated. (C) Frequency distribution of bite intervals, presented in terms of the absolute number of bites. Group mean plus standard error is indicated. Results from two-way ANOVA are displayed in top left corner of graph. Results from post-hoc test are displayed for data points where performance of WT and BACHD rats differed significantly. (D) Frequency distribution of bite intervals, presented in terms of the relative numbers of bites. Group mean plus standard error is indicated. Results from two-way ANOVA are displayed in top left corner of graph. Results from post-hoc test are displayed for data points where performance of WT and BACHD rats differed significantly.

(***) *p* < 0.001, ns (not significant)

# References

Clemensson, E. K. H., Clemensson, L. E., Fabry, B., Riess, O., and Nguyen, H. P. (2017). Further investigation of phenotypes and confounding factors of progressive ratio performance and feeding behavior in the BACHD rat model of Huntington disease. *PLoS One* 12, e0173232. Available at: https://doi.org/10.1371/journal.pone.0173232.

Friard, O., and Gamba, M. (2016). BORIS: a free, versatile open-source event-logging software for video/audio coding and live observations. *Methods Ecol. Evol.* 7, 1325–1330. doi:10.1111/2041-210X.12584.

Jansson, E. K. H., Clemens, L. E., Riess, O., and Nguyen, H. P. (2014). Reduced motivation in the BACHD rat model of Huntington disease is dependent on the choice of food deprivation strategy. *PLoS One* 9, e105662. doi:10.1371/journal.pone.0105662.

**
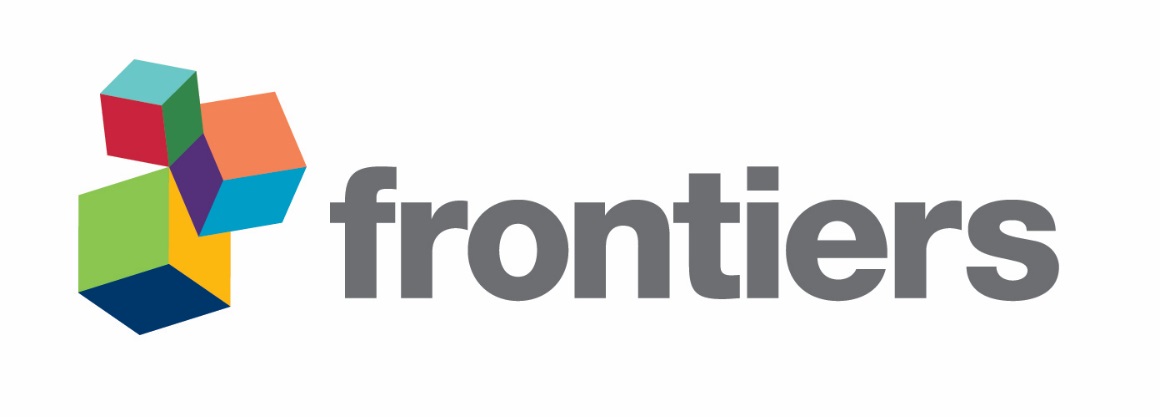
**
